# A systematic review of physician retirement planning

**DOI:** 10.1186/s12960-016-0166-z

**Published:** 2016-11-15

**Authors:** Michelle Pannor Silver, Angela D. Hamilton, Aviroop Biswas, Natalie Irene Warrick

**Affiliations:** 1 Department of Anthropology/Health Studies, University of Toronto Scarborough Campus, 1265 Military Trail, Toronto, Ontario M1C 1A4 Canada; 2 Institute of Health Policy, Management and Evaluation, University of Toronto, Toronto, Ontario Canada

## Abstract

**Background:**

Physician retirement planning and timing have important implications for patients, hospitals, and healthcare systems. Unplanned early or late physician retirement can have dire consequences in terms of both patient safety and human resource allocations. This systematic review examined existing evidence on the timing and process of retirement of physicians. Four questions were addressed: (1) When do physicians retire? (2) Why do some physicians retire early? (3) Why do some physicians delay their retirement? (4) What strategies facilitate physician retention and/or retirement planning?

**Methods:**

English-language studies were searched in electronic databases MEDLINE, Web of Science, Scopus, CINAHL, AgeLine, Embase, HealthSTAR, ASSA, and PsycINFO, from inception up to and including March 2016. Included studies were peer-reviewed primary journal articles with quantitative and/or qualitative analyses of physicians’ plans for, and opinions about, retirement. Three reviewers independently assessed each study for methodological quality using the Newcastle-Ottawa Scale for quantitative studies and Critical Appraisal Tool for qualitative studies, and a fourth reviewer resolved inconsistencies.

**Results:**

In all, 65 studies were included and analyzed, of which the majority were cross-sectional in design. Qualitative studies were found to be methodologically strong, with credible results deemed relevant to practice. The majority of quantitative studies had adequate sample representativeness, had justified and satisfactory sample size, used appropriate statistical tests, and collected primary data by self-reported survey methods.

Physicians commonly reported retiring between 60 and 69 years of age. Excessive workload and burnout were frequently cited reasons for early retirement. Ongoing financial obligations delayed retirement, while strategies to mitigate career dissatisfaction, workplace frustration, and workload pressure supported continuing practice.

**Conclusions:**

Knowledge of when physicians plan to retire and how they can transition out of practice has been shown to aid succession planning. Healthcare organizations might consider promoting retirement mentorship programs, resource toolkits, education sessions, and guidance around financial planning for physicians throughout their careers, as well as creating post-retirement opportunities that maintain institutional ties through teaching, mentoring, and peer support.

## Background

Over the last 40 years, across multiple jurisdictions, a pattern has emerged whereby a disproportionate number of physicians continue to practice beyond the traditional retirement age of approximately 65 years old [1, 2]. Accordingly, healthcare organizations often do not have effective succession strategies in place to manage their aging medical staff. The consequences of an older physician workforce can be dire and far-reaching. Replacing invaluable and experienced older physicians with trained but inexperienced younger physicians can be difficult [3]. In addition, the link between advancing age and deteriorating health may lead to increased medical errors, putting patient health at risk [4].

For an experienced physician, the decision regarding when to transition from practice to retirement can be about more than clinical [5] and technological competency [6], it can also involve internal emotional struggles. This is particularly the case when individuals have a strong sense of value attached to their work [7]. Evidence suggests that physicians’ adjustment to later career transitions can be facilitated by planning for retirement [8]. The objective of this review was to examine when physicians retire, why they retire early or delay retirement, and what strategies exist to facilitate physician retention and retirement planning. To our knowledge, no earlier studies have consolidated the literature with these questions in mind amidst the widespread call in the literature for such recommendations [9].

## Methods

The Preferred Reporting Items for Systematic Reviews and Meta-Analyses (PRISMA) guidelines were followed in the production and reporting of this systematic review [10].

### Study selection

Published articles were comprehensively searched using MEDLINE, Web of Science, Scopus, CINAHL, AgeLine, Embase, HealthSTAR, ASSA, and PsycINFO databases from inception up to and including March 2016. Our search strategy included the keywords “physician” and “retire” with all appropriate synonyms. All authors participated in the identification and final selection of studies.

### Study eligibility

**Fig. 1:**
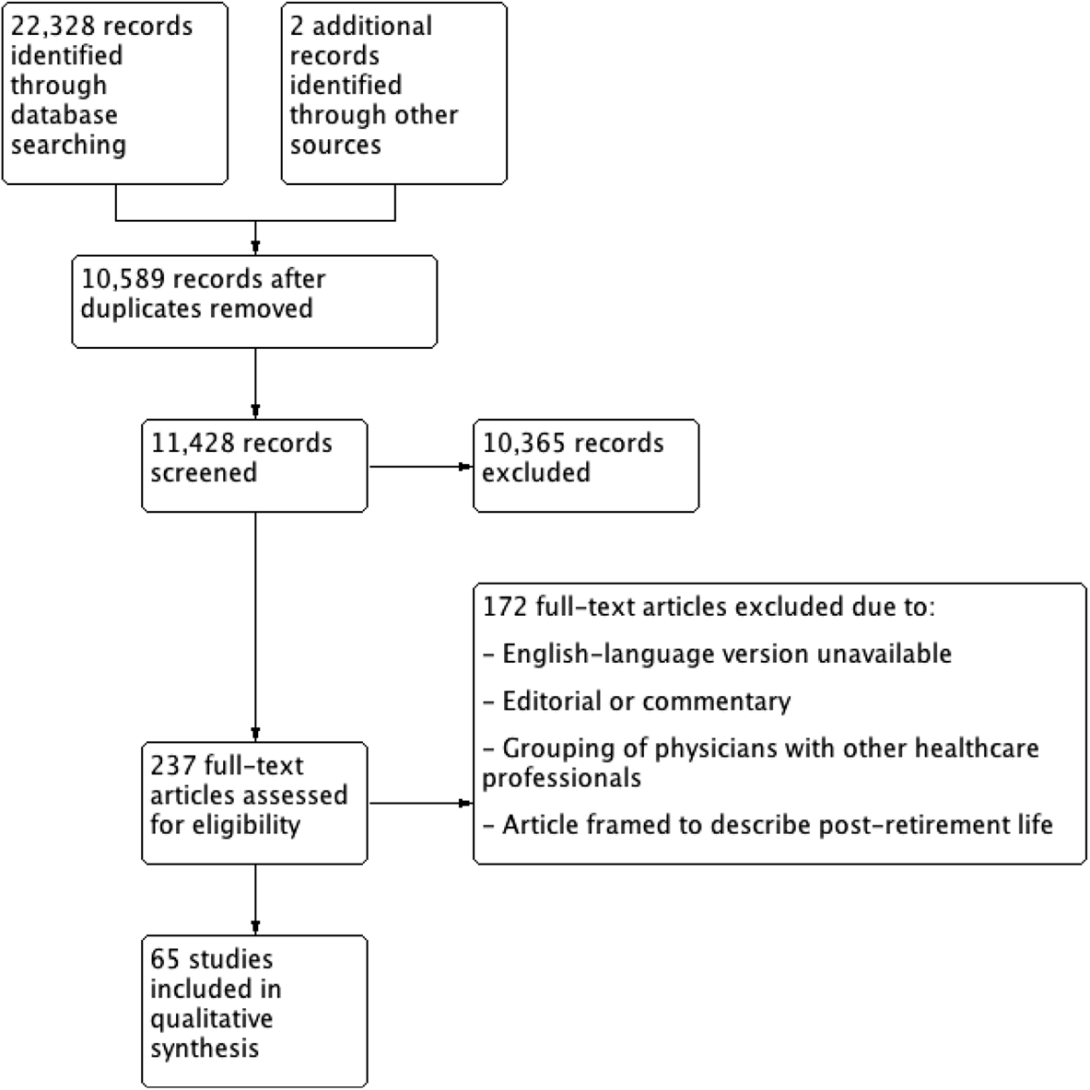
PRISMA flow diagram

The PRISMA flow diagram in Fig. 1 depicts the numbers of identified records, excluded articles, and included studies. Our inclusion criteria included published primary peer-reviewed journal articles with quantitative and/or qualitative analyses of physicians’ plans for, and opinions about, retirement. Excluded studies were non-primary research studies (editorials and commentaries), articles that grouped physicians with other healthcare professionals, or that only included dentists. After discussion, all authors agreed to constrain the search strategy to English-language articles, with no limitations on publication date up to March 2016. The search was supplemented by hand-searching the references of eligible studies and relevant review articles.

### Data extraction

The following information was extracted from qualifying studies: (i) geographic information, study design, data collection methodology, response rate, physician specialty; (ii) expected and actual retirement age; (iii) descriptive statistics related to demographic characteristics of the sample; and (iv) findings related to reasons for retiring, reasons for delaying retirement and obstacles to continued practice.

### Quality assessment

Three authors (ADH, AB, and NW) worked in pairwise rotation to independently review qualifying articles for methodological quality. The corresponding author (MPS) resolved any disagreements that could not be settled by consensus. We used the seven-item, Newcastle-Ottawa Quality Assessment Scale to assess the risk of bias for the 55 studies that had used quantitative methods [11]. The adapted Critical Appraisal of a Qualitative Study Tool from the Center for Evidence-Based Management was used to assess 10 studies that used qualitative methods [12]. All studies examined by quality assessment were given equal weighting and both quality assessment tools were selected on the basis of previously demonstrated reliability and validity when examining the views of healthcare professionals [13–15].

### Terminology

Early retirement is referred to in this study as either retirement that occurs earlier than the physician had planned [16] or to an exit from their profession at a relatively early age (i.e., younger than age 65) as compared to peers [17]. On time retirement refers to the conventional age of retirement, that is, at or around age 65 [18]. The literature commonly refers to retirement as late or delayed if physicians continue to work in a full-time capacity beyond the traditional age of retirement [19].

### Synthesis

Thematic analysis was used to identify and stratify concepts related to physician retirement timing into themes and subthemes [20]. Thematic analysis is an inductive qualitative data analysis process in which data are prepared, then organized using open coding to create categories and themes to build a conceptual understanding of a particular phenomenon and analyze the meaning of data within their particular context [21].

## Results

### Study characteristics

Table 1 summarizes the characteristics of the 65 studies included in this review. The studies were published between 1978 and 2015, with 33 studies based in the United States, others in Australia, Canada, Finland, Israel, Netherlands, New Zealand, the United Kingdom, and one across 20 countries of high-, medium-, and low-income economies. A variety of practicing and retired physicians were sampled with a range of specializations from general and multidisciplinary physicians to anesthesiologists, dentists, general and specialist surgeons, obstetrician-gynecologists, otolaryngologists, ophthalmologists, pediatricians, psychologists, radiologists, and urologists.

**Table 1:** Characteristics of included studies

Study	Location	Study method (source, if not self-administered)	Sample size (response rate)	Participants (average age and/or age range)
Anderson [37]	United States	Survey (administered by the American Medical Colleges and the American College of Obstetricians and Gynecologists)	< Age 50, 2000 (40.3%) > Age 50, 2100 (57.3%)	Obstetrician-gynecologists (average age <50 was 44 years, average age >50 was 65 years)
Austrom [58]	United States	Survey (modified version of American Association of Orthopaedic Surgeons survey)	1834 (43%)	Multidisciplinary physicians and spouses (average age 75 years)
Baker [56]	United States	Survey	500 (46%)	Psychiatric physicians (age 50 to 69 years)
Baker [59]	United States	Survey	125 (53%)	Black psychiatrists (age 31 to 74 years)
Baker and Hishinuma [74]	United States	Survey	AMA: 187 (58%); NMA: 85 (65%)	Multidisciplinary physicians. AMA members (age 50 years or older), NMA members (age 30 years or older)
Batchelor [22]	United States	Survey/interviews	20 (80%)	Senior women physicians (age 59 to 95 years)
Bieliauskas [75]	United States	Computerized cognitive test/survey	359 (82%)	Surgeons (age 45 or older, average age 61.4 years)
Brett [51]	Australia	Survey	281 (59%)	Multidisciplinary physicians (age 45 to 65, average age 52.4 years)
Burke [76]	United Kingdom	Administrative data, Department of Health and f Insurance industry (the Dentists’ Provident Society)	393(N/A)	Retired dentists (N/A)
Chambers [69]	United Kingdom	Survey	348 (72%)	Multidisciplinary physicians (average age 55 years)
Crowson [6]	United States	Retrospective study (Duke University Hospital Department of Human Resources)	208	Multidisciplinary physicians (average age between 45 and 48.1)
Davidson [77]	United Kingdom	Survey	2398 (78%)	Multidisciplinary physicians (average age mid-40s)
Davidson [52]	United Kingdom	Survey	1460 (85%)	Multidisciplinary physicians (average age 48 years)
Deitch [48]	United States	Survey (ACR Committee on Manpower)	2804 (69%)	Radiologists, radio-oncologists, and nuclear medicine specialists (average age in years <35 (11%), 35 and 44 (37%), 45 and 54 (32%) and 55 or older (20%).
De Santo [78]	Algeria, Australia, Brazil, Egypt, France, Germany, Greece, Italy, Malta, Libya, Poland, Romania, Slovak Republic, Slovenia, Switzerland, The Netherlands, Tunisia, Turkey, UK and USA	Survey	113 (89.1%)	Active professors and emeritus/retired professors from 99 departments of medicine/universities worldwide (NA)
Dodds [46]	United States	Survey	96 (82%)	Academic chairs of ophthalmology departments (age range <50 to >70, average age 58 years)
Donner [79]	United States	Review of data based on survey (ACR Commission on Human Resources, 2012 and 2013)	N/A	Radiologists
Draper [40]	Australia and New Zealand	Survey	281 (60%)	Psychiatrists (ages 55–87 and average age 65.5 years)
Draper [80]	Australia and New Zealand	Survey (respondents were fellows of the Royal Australian and New Zealand College of Psychiatrists resident in Australia or New Zealand)	529 (57.9%)	Psychiatrists (age 40 years and older)
Eagles [30]	United Kingdom	Survey	180 (50%)	Consultant psychologists (N/A)
Evans and Ghosh [43]	United States	Survey	749 (17%)	Headache medicine specialists
Farley [39]	United States	Survey (American Academy of Orthopaedic Surgeons in cooperation with the Association of American Medical Colleges Center for Workforce Studies)	3001 (33.5%)	Orthopedic surgeons (age 50 years and older)
Fletcher and Schofield [38]	Australia	Data from the Australian Institute of Health and Welfare (AIHW) Medical Labour Force Survey from 1995 to 2003	N/A	Psychiatrists (age 50 years and over)
Florence [81]	United States	Survey	785(22%)	Transplant surgeons (average age 48.7 years)
French [36]	United Kingdom	Survey	2923(61%)	Consultants and specialists (average age 47 years)
French [23]	United Kingdom	Survey/interviews/focus groups	924 (50%)	Multidisciplinary physicians (average age 43 years)
Gee [82]	United States	Telephone interview (Gallup Poll)	451 (89%)	Urologists (age in years <36 (9%), 37 to 45 (29%), 46 to 54 (30%), 55 to 64 (25%), <65 (7%))
Goldberg [57]	United States	Survey of American College of Emergency Physicians members (two separate mailings in the fall of 2006 and winter of 2007)	1000 (80%)	American College of Emergency Physicians members over the age of 55 years (average age 57 years)
Grauer and Campbell [50]	Canada	Survey	58 (53.7%)	Multidisciplinary physicians (average age 71.2 years)
Greenfield and Proctor [83]	United States	Survey	659 (75%)	Surgeons (age in years <50 (7%), 50–60 (29%), 60–70 (35%), >70 (28%)
Gregory and Menser, [63]	United States	Longitudinal (three wave) online survey	97, 91, 56 (65.5%, 54.9%, 58.4%), respectively	Primary/ambulatory care physicians (N/A)
Grondin [61]	Canada	Survey	97 (71%)	Thoracic surgeons (average age 47.7 years)
Hall [5]	United States and Canada	Survey	1444 APS members (35%); 148 pediatric department chairs (40%)	Senior pediatricians and pediatric department chairs (ages 39 to 94, average age 65 years)
Heponiemi [44]	Finland	Survey (Finnish Health Care Professional Study)	1393 (27.9%)	Multidisciplinary physicians (ages 45 to 65 years)
Hill [24]	United Kingdom	Semi-structured interviews/survey	23 (N/A)	Dentists (NA)
Jacobson and Eran [25]	Israel	Interview	317 (89.5%)	Multidisciplinary physicians (age 50 years or older)
Jonasson and Kwakwa [84]	United States	Survey	373 (84%)	General surgeons (NA)
Joyce [42]	Australia	Longitudinal survey (Medicine in Australia: Balancing Employment and Life, Cycles 2009 to 2012)	1073 (2009, 82.64%, 2010, 82.24%, 2011, 75.51% and 2012, 75.32%)	Physicians and specialists aged ≥65 years
Kendell and Pearce [85]	United Kingdom	Survey	173(82%)	Consultant psychiatrists (NA)
Landon [49]	United States	Data for this study are from the first 2 rounds of the Community Tracking Study (CTS) Physician Survey	16,681 (63%)	Primary care and specialist physicians initially spending at least 20 h/week in direct patient care activities were studied (average age 47.5 years for practicing and 63.0 years for retired physicians)
Lee [86]	United States	Telephone interview/survey	33 (75%)	Multidisciplinary rural physicians (age 60 years or older)
Lee [87]	United States	Survey	995 (N/A)	Surgeons (age in years <35 (13.37%), 35–44 (12.96%), 45–54 (18.69%), 55–65 (31.06%), >65 (23.92%))
Luce [7]	United Kingdom	Survey	518 (72.5%)	Multidisciplinary physicians (age 45 years or older)
Moriarty [88]	United States	Survey sent to all members of the American College of Radiology (ACR), the Association of University Radiologists (AUR), and the Society of Chairs of Academic Radiology Departments (SCARD)	~37900 (11%)	Practicing radiologists (NA)
McGuirt and McGuirt [89]	United States	Survey	438 (31.5%)	Otolaryngologists (ages 40 to 80, average age 63.2 years)
Mears [41]	United Kingdom	Survey	835 (59%)	Consultant psychologists (age 50 years or older)
Meghea and Sunshine [54]	United States	Survey (American College of Radiology’s 2003 Survey of Radiologists)	1676 (63%)	Radiologists (ages 35 to 75 years)
Newton [26]	United Kingdom	Semi-structured interviews	21 (N/A)	Multidisciplinary physicians (age 44 years or older)
Onyura [19]	Canada	Secondary analysis of data from a larger study on issues of late-career planning among academic physicians; semi-structured interviews	21	Academic physicians at a Canadian medical school (n = 21, average age = 63 years, age range = 46–72 years)
Orkin [34]	United States	Survey	8670 (37.2%)	Anaesthesiologists (age 50–79 years, average age 60.1 years)
Peisah [45]	Australia, Canada, United States	Semi-structured interviews	25 (N/A)	Multidisciplinary physicians (aged 60 or older, average age 67.5 years, age range = 60–88 years)
Pit and Hansen [16]	Australia	Survey	92(56%)	Multidisciplinary physicians (average age 51 years)
Quandango [27]	United States	Semi-structured interviews	40 (N/A)	Multidisciplinary physicians (ages 55 to 72)
Rayburn [31]	United States	American Medical Association Master file	N/A	Obstetrician-gynecologists
Reuben and Silliman [47]	United States	Survey	282 (70%)	Multidisciplinary physicians (age 65 or older, average age 71 years)
Rittenhouse [33]	United States	Survey	967 (N/A)	Multidisciplinary physicians (<55 years, 62.8%, 55–64 years, 27.3%, >65 years, 9.9%)
Rowe [90]	United States	Survey	169 (84%)	Physicians (52–96 years)
Sansom [28]	England	Semi-structured interviews	23	General practitioners (50–60 years)
Shanafelt [53]	United States	Survey, American Society of Clinical Oncology	2998 (49.7%)	US oncologists
Sibbald [32]	United Kingdom	Survey	1949 (N/A)	Multidisciplinary physicians (average age 55 years)
Silver [29]	Canada	Focus groups	16	Academic physicians over 50 years old within the Department of Medicine at the University of Toronto
Smith [91]	Canada	National survey was administered to all Canadian otolaryngologists	65 (65%)	Otolaryngologists who were identified to have a clinical practice composed of >50% rhinology (average age: 46 years)
Sutinen [35]	Finland	Survey	819 (55%)	Multidisciplinary physicians (ages 26 to 63 years)
Van Greuningen [17]	Netherlands	Retrospective survey (2 waves)	520 (60%); 405 (54%)	Self-employed general practitioners retired before age 65
Wakeford et al. [18]	United Kingdom	Interview	250 (79%)	Multidisciplinary physicians (average age: 61.4 years)

Tables 2 and 3 summarize the quality of the included studies. Qualitative studies [9, 18, 22–29] were found to be methodologically strong, with credible results deemed relevant to practice. The majority of quantitative studies had adequate sample representativeness (76% of studies), had justified and satisfactory sample size (89% of studies), used appropriate statistical tests (59% of studies), and collected primary data by self-reported survey methods (91% of studies). Studies were rated poorly on the ascertainment of exposure (i.e., how the outcome of interest was obtained either by secure record, structured interview, or self-reported) due to the use of non-validated measurement tools (51% of studies). Nearly half (49%) of the studies were rated poorly for comparability since they did not control for any potential confounders.

**Table 2:** Assessment of studies included in this review using the Newcastle-Ottawa Quality Assessment Scale for cohort studies as well as the adapted version for cross-sectional studies

	Selection ^a^				Comparability ^b^	Outcome ^c^		Quality score
37	Representativeness of sample	Sample size	Non-respondents	Ascertainment of exposure		Assessment of outcome	Statistical test	
Anderson [47]	A	A	B	C	A	C	A	6
Austrom [58]	B	A	A	B	–	C	A	6
Baker [56]	A	A	A	C	–	C	B	4
Baker [59]	A	A	A	B	–	C	B	5
Baker and Hishinuma [74]	B	A	B	B	A/B	C	A	7
Batchelor [22]	C	B	B	B	–	C	B	2
Biellauskas [75]	B	B	C	A	A/B	C	A	6
Brett [51]	B	B	B	B	–	A	A	5
Burke [76]	C	A	C	B	–	C	B	3
Chambers [69]	A	A	A	A	–	C	A	6
Crowson, [6]	A	A	A	A	A	B	–	7
Davidson [77]	A	A	A	C	–	C	A	5
Davidson [52]	A	A	C	B	A	C	A	6
Deitch [48]	A	A	A	B	A/B	C	A	8
De Santo [78]	A	A	B	B	–	C	B	4
Dodds [46]	A	A	A	A	A/B	C	A	9
Donner [79]	D	C	C	C	–	D	B	0
Draper [40]	A	A	A	B	A	C	A	7
Draper [80]	A	A	B	A	A/B	C	A	8
Eagles [30]	A	A	B	B	A	C	B	5
Evans and Ghosh [43]	A	B	B	A	–	C	A	5
Farley [39]	A	A	B	A	–	C	B	4
Fletcher and Schofield [38]	A	A	C	A	A/B	C	A	8
Florence [81]	A	A	B	B	–	C	B	4
French [36]	A	A	A	A	A	C	A	8
French [23]	A	A	A	A	A	C	A	8
Gee [82]	A	A	B	B	–	C	A	5
Goldberg [57]	A	A	B	A	–	C	A	6
Grauer and Campbell [50]	D	B	C	B	–	C	B	2
Greenfield and Proctor [83]	A	A	B	B	A	C	B	5
Gregory and Menser [63]	B	A	B	A	A	C	A	7
Grondin [61]	A	A	B	A	–	C	A	6
Hall [5]	A	A	B	B	–	C	B	4
Heponiemi [44]	A	A	B	A	A/B	C	A	8
Hill [24]	C	A	C	B	–	C	B	3
Jacobson and Eran [25]	A	A	B	A	A/B	C	A	8
Jonasson and Kwakwa [84]	A	A	B	B	A	C	B	5
Joyce [42]	A	A	A	C	A	C	B	8
Kendell and Pearce [85]	A	A	B	C	–	C	B	3
Landon [49]	B	A	A	B	A/B	C	A	8
Lee [77]	A	A	A	B	–	C	B	5
Lee [87]	B	A	B	B	–	C	A	5
Luce [7]	A	A	B	A	–	C	A	6
Moriarty [88]	A	B	B	B	A/B	C	B	5
McGuirt and McGuirt [89]	B	A	B	B	–	C	B	4
Mears [41]	A	A	B	B	A	C	A	6
Meghea and Sunshine [54]	A	A	A	B	A/B	C	A	8
Newton [26]	C	A	B	A	–	C	B	4
Onyura [19]	B	A	C	B	–	C	B	4
Orkin [34]	A	A	B	B	A/B	C	A	7
Peisah [45]	C	A	C	A	–	C	B	4
Pit and Hansen [16]	B	A	B	A	A/B	C	A	8
Quandango [27]	C	A	B	B	–	C	B	3
Rayburn [31]	A	A	B	B	–	B	B	5
Reuben and Silliman [47]	A	A	A	B	A/B	C	A	8
Ritternhouse [33]	A	A	A	B	A/B	B	A	9
Rowe [90]	A	A	B	C	–	C	B	3
Shanafelt [53]	A	A	A	A	A/B	C	A	9
Sibbald [32]	A	A	A	A	A/B	A	A	9
Silver [29]	B	A	B	B	–	C	B	4
Smith [91]	A	A	C	A	–	C	B	5
Sutinen [35]	A	A	A	A	A/B	C	A	8
Van Greuningen [17]	A	A	B	A	–	C	A	7
Wakeford [18]	A	A	C	B	–	C	B	4

“–”, not reported.

Wells, G.A.; Shea, B.; O’Connell, D.; Peterson, J.; Welch, V.; Losos, M.; Tugwell, P. The Newcastle-Ottawa Scale (NOS) for assessing the quality of nonrandomized studies in meta-analyses. Available online: http://www.ohri.ca/programs/clinical_epidemiology/oxford.asp

^a^Selection (5 points in total): (1) *Representativeness of the sample*: A, truly representative of the average in the target population (1 point); B, somewhat representative of the average in the target population (1 point); C, selected group of users (no points); D, no description of the sampling strategy (no points). (2) *Sample size*: A, justified and satisfactory (1 point); B, not justified (no points). (3) *Non-respondents*: A, comparability between respondents and non-respondents characteristics is established, and the response rate is satisfactory (1 point); B, the response rate is unsatisfactory, or the comparability between respondents and non-respondents is unsatisfactory (no points); C, no description of the response rate or the characteristics of the responders and the non-responders (no points). (4) *Ascertainment of the exposure*: A, validated measurement tool (2 points); B, non-validated measurement tool, but the tool is available or described (1 point); C, no description of the measurement tool (no points).

^b^Comparability (2 points in total): (1) *Confounding factors are controlled*: A, the study controls for the most important factor (1 point); B, the study control for any additional factor (1 point).

^c^ Outcome (3 points in total): (1) *Assessment of the outcome*: A, independent blind assessment (2 points); B, record linkage (2 points); C, self-report (1 point); D, no description (no points). (2) *Statistical test:* A, the statistical test used to analyze the data is clearly described and appropriate, and the measurement of the association is presented, including confidence intervals and the probability level (*P* value) (1 point); B, the statistical test is not appropriate, not described or incomplete (no points).

**Table 3:** Assessment of qualitative studies included in this review

	Batchelor, 1990 [22]	French et al., 2006 [23]	Hill et al., 2010	Jacobson and Eran, 1980 [24]	Newton et al., 2004 [26]	Peisah, Gautam, and Goldstein, 2009 [9]	Quandango, 1978 [27]	Sansom, 2016 [28]	Silver, Pang, and Williams, 2015 [29]	Wakeford, Roden, and Rothman, 1986 [18]
1. Does the study address a clearly focused question/issue?	Y	Y	Y	Y	Y	Y	Y	Y	Y	Y
2. Is the research method (study design) appropriate for answering the research question?	Y	Y	Y	Y	Y	Y	Y	Y	Y	Y
3. Was the context clearly described?	Y	Y	Y	Y	Y	Y	Y	Y	Y	Y
4. How was the fieldwork undertaken? Was it described in detail? Are the methods for collecting data clearly described?	Y	Y	Y	Y	Y	N	Y	Y	Y	N
5. Could the evidence (fieldwork notes, interview transcripts, recordings, documentary analysis, etc.) be inspected independently by others?	N	Y	Y	N	Y	Y	Y	Y	Y	N
6. Are the procedures for data analysis reliable and theoretically justified? Are quality control measures used?	N	Y	Y	N	Y	N	Y	Y	Y	N
7. Was the analysis repeated by more than one researcher to ensure reliability?	N	Y	Y	Y	Y	N	N	Y	Y	N
8. Are the results credible, and if so, are they relevant for practice?	Y	Y	Y	Y	Y	N	Y	Y	Y	Y
9. Are the conclusions drawn justified by the results?	Y	Y	Y	Y	Y	Y	Y	Y	Y	Y
10. Are the findings of the study transferable to other settings?	Y	N	Y	Y	Y	N	Y	Y	N	N

Responses in the affirmative (Y) are indicative of higher validity and quality; those in the negative (N) indicate absence of support.

Adapted from Crombie, *The Pocket Guide to Critical Appraisal*; the critical appraisal approach used by the Oxford Centre for Evidence Medicine, checklists of the Dutch Cochrane Centre, BMJ editor’s checklists and the checklists of the EPPI Centre.

### Physician retirement age

Physicians’ actual retirement age and their intended or planned retirement age are distinguished in Table 4. Physicians’ intended or planned retirement age refers to the age they speculate they will most likely be when they reach retirement [30]. This differs from physicians’ actual retirement age, represented by the chronological age at which they reported being fully retired [31]. Comparisons of on-time, early, and delayed retirement were made in a context relative to physician peers [16, 32, 33] and across subspecialties [31, 34]. In some instances, comparisons were made to other professional groups such as social workers [35].

**Table 4:** Expected and actual physician retirement age

	50–59 years	60–69 years	>70 years	“Never”
Expected retirement age	Burke [76] *Eagles* [30] Luce [7] Fletcher [38] Mears [41] Goldberg [57] Sansom [28]	*Anderson* [37] Dietch [48] Dodds [46] Farley [39] Florence [81] Grondin [61] Mears [41] *French* [36] French [23] Gee [82] Pit [45] Rayburn [31] *Shanafelt* [53] Smith [91] *Wakeford* [18]	Batchelor [22]	Draper [40]
Actual retirement age	Baker [52] *Eagles* [30] Sansom [28]	*Anderson* [37] Austrom [58] Batchelor [22] Farley [39] Fletcher [38] *French* [36] Jonasson [84] Meghea [54] Luce [7] Orkin [34] Rayburn [31] Rowe [90] Van Greuningen [17] *Wakeford* [18]	Joyce [42] Rayburn [31]	–

Note: Average or highest reported retirement ages are reported.

Studies where the majority of physicians met retirement age expectations are in italics

Our findings suggest the average age for actual and expected retirement was commonly reported to be between 60 and 69 years, respectively. Several studies [7, 18, 22, 30, 31, 36–39] examined the age that physicians expected to retire, and the age they actually retired (underlined in Table 4). The actual retirement age was found to be consistent with their expected retirement in all studies where the actual and expected retirement ages were jointly reported. These studies highlight that a variety of methods are used to determine usual age at retirement and that physicians’ retirement intentions can, but not always, translate into actual retirement behaviors.

### Reasons for retiring early and obstacles to practice

Common reasons for retiring early included low job satisfaction, medicolegal issues, health concerns, and financial troubles. Low job satisfaction involved perceptions of low job control, low morale, and dissatisfaction with the internal justice system of medicine as a self-regulated profession [5, 9, 28, 40]. This disillusionment was expressed by a sense of frustration with colleagues [27, 35], feeling undervalued, lacking prestige [16, 41], and a loss of interest in their work [10]. Excessive workload [17, 42] and burnout were associated with intentions to retire [28, 43]. Medicolegal issues often arose from a lack of satisfaction with the regulation of medicine for reasons of unwelcome change, bureaucracy, oppressive management [26, 35, 44], and issues with physician partners [26, 45]. Experiencing poor health, cognitive decline, difficulty sleeping, and psychological distress were also factors leading to a physician’s retirement [15, 18, 19, 34, 36, 38, 46–50].

The decision to retire early was also linked to preserving one’s health to lead a healthy retirement [51, 52]. Financial issues contributing to a physician’s early retirement included: increasing costs of retaining a practice, malpractice costs, and other economic pressures [5, 25, 37, 39, 47, 52], insufficient financial remuneration, and pension security [7, 46, 52, 53]. However, one study [42] found that retirement was not associated with perceived adequacy of finances, or general health status. Several studies noted that physicians working in institutions or in countries where the policy landscape changed considerably were more inclined to retire in part due to poor work satisfaction that resulted from changing circumstances around the delivery of care and doctoring regulations [29]. Table 5 summarizes the obstacles related to continuing practice.

**Table 5:** Obstacles to practice

Subtheme	Study
Workplace frustration: bureaucracy, accreditation, healthcare reform, demands from government, alienation by changes to working life, low job control, low organizational justice, poor teamwork and workforce shortages	Brett [51]; Crowson [6]; Evans [43]; Fletcher and Schofield [38]; Heponiemi [44]; Hill [24]; Kendell and Pearce [85]; Lee [87]; McGuirt and McGuirt [89]; Mears [41]; Newton [26]; Sansom [23]; Sutinen [35]; Van Greuningen [17]
Workload pressures: patient demands, long hours, demanding on-call schedules and sacrifice of family/free time, work-life balance	Brett [51]; Chambers [69]; Draper [80]; Evans [43]; French [36]; Joyce [42]; Mears [41]; Meghea and Sunshine 549]; Newton [26]; Goldberg [57]; Sibbald [32]; Onyura [19]; Sansom [28]; Shanafelt [53]; Van Greuningen [17]
Career dissatisfaction: lost interest in work	Brett [51]; Chambers [69]; Crowson [6]; Evans [43]; Hill [24]; Joyce [42]; Luce [7]; Orkin [34]; Ritternhouse [33]; Sibbald [32]; Landon [49]; Van Greuningen [17]
Health: excessive stress, health and mental health concerns (thoughts of suicide, emotional exhaustion), and spousal health	Dodds [35]; Draper [80]; Goldberg [57]; Hall [5]; Hill [24]; Luce [7]; Newton [26]; Pit and Hansen [16]; Sansom [28]; Van Greuningen [17]
Finances: pension, economic concerns, costs of continuing to practice, retirement not being written into partner agreements, general guidance, insurer policies affecting payment	Evans [43]; French [36]; Grondin [75]; Hall [5]; Lee [78]; Orkin [34]; Sansom [28]; Van Greuningen [17]; Wakeford [18]
Skills and competencies: worry over competencies amidst technological advancements and new modalities of diagnosis or treatment	Crowson [6]; Draper [80]; Goldberg [57]; Grauer and Campbell [50]; Hall [5]; Sansom [28]

### Reasons for delaying retirement

Reasons for physicians delaying retirement included being satisfied with their career [16, 34, 37, 39, 47–49], institutional flexibility [51], a feeling of responsibility for their patients [18, 19, 37, 38, 47, 51], a desire to be healthy and keep being active [18, 34, 44, 46], financial reasons [7, 34, 36, 39, 46, 47, 50, 53, 54], and a lack of interests outside of medicine [46]. In particular, institutional flexibility was a positive driver of physicians’ work satisfaction and their desire to remain in practice as they were provided reasonable access to sabbaticals, flexible working hours, and control over their job and career development [7, 39, 51, 55].

The continuation of medical practice is deeply rooted in a desire to keep active and focus on the social and intellectual elements of continuing to practice [46, 47, 56]. Physicians expressed concerns over their decision to retire, due to fear of losing their primary identity or purpose [9, 19, 50, 57], or being uncomfortable with the methods used to enforce their retirement [58]. Retirement concerns also stemmed from personal issues such as a fear of potential changes in the relationship with their spouse following retirement [58], a fear of excessive leisure time and lack of hobbies [50], and inadequate financial preparation for retired life [34, 57]. Several studies also pointed to a link between physicians’ restricted availability of free time and the development of external hobbies or interests. Nonetheless, continuing in medicine was viewed as a better alternative to life in retirement [52, 56, 59].

### Strategies to facilitate physician retention and retirement planning

Key strategies to facilitate physician retention and retirement planning included offering flexible work hours, minimal work barriers, enhancing work satisfaction, prioritizing physician health, and attention to finances. In particular, options such as part-time employment and less bureaucracy were suggested as ways to facilitate a working environment that would be amenable to physicians overburdened by work demands in ways that might foreshorten their career. In addition, providing opportunities for professional development to help physicians develop or change the content of their work was offered as an important means of retention, as well as a mechanism for making successful later career transitions out of medicine. Attention to personal matters such as physicians’ own health and finances in ways that reduced work-related stress or protected physicians’ income through pension plans were also important in enhancing physician retention and enticing continued practice. Table 6 summarizes the retention schemes described by the studies included in this review.

**Table 6:** Retention schemes

Subtheme	Study
Flexible work hours: part-time employment options, gradual reduction, flexible hours or sabbatical, decreased on-call, relief of workload pressure	Anderson [37]; Brett [51]; Davidson [52]; Eagles [30]; French [36]; French [23]; Hall [5]; Jacobsen and Eran [25]; Newton [26], 2004; Goldberg [57]
Minimal work barriers: less bureaucracy, increased staff, improved working conditions, support to maintain/update competencies, more time with patients	Brett [51]; Davidson [52]; Eagles [30]; Kendell and Pearce [85]
Work satisfaction: professional/clinical freedom, attend conferences and rounds, office space, chances to develop or change content of their work (i.e., teaching opportunities)	Brett [51]; Chambers [69]; Eagles [30]; Farley [39]; Hall [5]; Landon [49]
Health: continuing good or better than expected health at expected retirement age, strategies to reduce work-related stress, support prioritizing health	Brett [51]; Davidson [52]; Draper [80]; Luce [7]; Pit and Hansen [16]
Finances: protected pensions, being highly paid, financial necessity	Brett [51]; Davidson [52]; Eagles [30]; French [36]; Hall [5];

## Discussion

Our review confirmed that physicians are likely to remain in their practice beyond the traditional retirement age of 65. To put these results into context, it is worthwhile to first consider that in recent decades, workers are generally tending toward later retirement. While a person aged 50 in the workforce during 1997 was expected to continue working 13 more years, an average worker of the same age in 2009 was expected to work an additional 3 years, eventually retiring at an age of 66 on average [60]. This systematic review illustrates that the average age physicians expect to retire lies closest to age 60 while their age at actual retirement is closer to 69. This represents an average of 3 years later than the general population.

Retirement trends have been shifting over the last few decades in response to an increasing lifespan, adjustments to economic market fluctuations, and concerns about the sustainability of social security entitlements [61, 62]. In particular, concerns about economic market fluctuations are particularly relevant for physicians who tend not to have access to group pension funds that other workers, such as teachers or health-care administrators, might have access to. We found delayed retirement among physicians is likely to be influenced by flexibility and intensity of working hours, work satisfaction, career opportunities, resource adequacy, intrinsic value, convenience, financial incentives, and relations with co-workers. As one might expect, these are many of the same determinants that impact retirement among other professionals. However, it is also likely that other factors such as attachment to work and strong work identity may serve as an additional rationale for working beyond the traditional retirement age [29]. Furthermore, it is likely that the advanced training and late entry into the work force also renders physicians more likely to retire later than the average worker.

Physician’s early retirement, like that of other professions, is often brought about by negative dimensions of work satisfaction. Where physicians may differ from the general population is in the complex nature of their work, which involves a unique combination of advanced training, autonomy, skill, experience, leadership, and decision-making that can have life or death consequences. Many studies have examined the implications of physician burnout [43, 63], thus suggesting that physicians face unique challenges as it relates to extending their careers. Physicians’ early retirement is an important concern as other research attests to the risks to patient care associated with physician shortages [64]. While the studies examined in this review did not highlight gender as an important factor relevant to early retirement, there is evidence to indicate that there are high burnout rates among women physician [65]. This is likely to influence physician retirement rates in ways that were not captured in the studies reviewed here, and relevant given than women live longer than men on average and are increasingly entering medicine.

Successful retirement planning was found to be related to being prepared for the financial demands, physical changes, and psychosocial dynamics associated with aging and leaving the workforce, consistent with prior research based on the general population [66–68]. Findings based on these studies of physicians suggest that a reduction of working hours may present as one of the most successful instruments for staff retention [17]. A shift toward non-clinical duties such as teaching and mentorship may also help with retention [42] and also facilitate knowledge transfer to younger professionals. The theory of purposeful work behavior [59] posits that, when job characteristics act in concert with individuals’ motivational striving, psychological meaningfulness may be gleaned from their work. Thus, if physicians are given opportunities to pursue preferred work tasks such as teaching over clinical rounds [30, 69], then their experiences of greater meaningfulness in their work may trigger task-specific motivation [70]. This can result in a willingness to continue working in hospital settings in a way that benefits the enterprise as a whole.

On the whole, health was also shown to be an important factor determining whether physicians chose to remain in the workforce. Excessive workload and poor health were found to be major reasons a physician may choose to retire. As such, healthcare organizations may consider strategies that improve physician health by addressing the physical fitness and risk-related habits of physicians. Some potential interventions might include fostering a culture that is supportive of taking sick days [71] along with proper mechanisms that allow physicians not to overburden one another when taking sick days. Findings from most of the studies included in this review also indicate that a supportive and highly satisfying work environment facilitates physician retention.

Organizations can have a role in facilitating the graceful and timely exit of the well-established physician but should exercise caution that the approach taken is not driven by ageist stereotypes or leading to feelings of being “pushed out” [26]. Physicians retiring beyond the traditional retirement age will have accumulated decades of knowledge and experience, and offer an invaluable resource to the medical enterprise [42]. The challenge is that, without foresight of the timing associated with physicians’ plans for retirement, institutional hospital succession plans come to a halt. The timing of physician retirement becomes particularly salient, not only for human resources planning but also for patient care continuity and transitions of care in hospital enterprises where mentors of the younger hospitalist workforce may be scarce [3]. In this way, the medical enterprise must strike a delicate balance between encouraging preparation for retirement and delaying the timing and eventual transitions of its most experienced staff who will be replaced by a growing pool of younger physicians who stand waiting in the wings for professional opportunities.

### Limitations and recommendations for future research

Research on the factors that influence physician retirement timing and planning for retirement is still in its early stages, and future exploration into the most promising interventions is needed to further delineate our preliminary findings. Some limitations of this review include the restriction to English language studies, which excludes the perspectives of physicians from non-English speaking regions. Furthermore, our analysis is based on a heterogeneous sample of physicians spanning across diverse specializations, with jurisdictional differences in regulations, mandatory retirement legislation, pension systems, and differences in remuneration across healthcare systems. In addition, because the studies examined in this review used a cross-sectional design and were limited in terms of the types of analyses they performed, we were unable to perform a meta-analysis of the included studies. Furthermore, our search was restricted to peer-reviewed literature, thus future research may enhance the findings of this study by examining the grey literature on this topic. Future studies can also benefit from exploring the healthcare context in which the physicians practice, gender differences as they relate to physician retirement planning and physicians’ transitions from practice, and consider following physicians over time to better understand factors that facilitate planning for a transition from practice.

The abolition of policies of mandatory retirement across many countries has encouraged some physicians to extend their medical careers, generating greater unpredictability in later career transitions [5, 31]. While several attempts, including our own study, have aimed at improving understanding of health workforce issues and implications of aging and timing of physicians’ work, future policy research should continue forecasting physician retirement trajectories and human resource strategies in ways that can account for older physicians who want to remain in clinical practice beyond traditional retirement age [17, 72, 73]. Recommendations for next steps in policy reform at the organizational and health system level may come from within hospital and other related organizations which aim to address intentions to leave by improving psychosocial working conditions for the medical profession [64] and scaling back workloads to retain the best talent in experienced physicians [42]. Notably, healthcare managers may pursue recommendations for an “integrated” approach to recruitment, retention, and retirement planning that aids in better anticipating upcoming retirement transitions, shifts cultural attitudes toward retirement planning, and brings together a larger strategy to ameliorate succession planning.

## Conclusions

Knowledge of when physicians plan to retire and how they can transition out of practice has been shown to aid effectual succession planning. This paper identified that the most common age of retirement for physicians was between 60 and 69. We examined the literature on reasons for early and delayed retirement, as well as strategies shown to be effective in supporting continuing practice. We found excessive workload, poor health, and low job satisfaction to be major reasons for why a physician may choose to retire early. Delayed retirement or reasons physicians’ work lives were extended was explained by financial obligations, strong work identity, career satisfaction, and institutional flexibility. Strategies that supported continuing to practice included offering flexible work hours, minimal work barriers, enhancing work satisfaction, prioritizing physician health, and attention to finances. As this line of inquiry is still developing, we recommend future research and strategies consider the impact of a physician’s flexible work hours, gradual reduction in responsibilities, and the ways in which resources for financial planning facilitate physician retirement planning. In addition, an important component of successful retirement planning concerns the creation of meaningful activity after retirement [31]; thus, healthcare organizations should consider promoting retirement resource toolkits, education sessions, and guidance around financial planning for physicians throughout their careers, as well as creating post-retirement opportunities that maintain institutional ties through teaching, mentoring, and peer support [68, 69]. Preparation for a retirement that is tailored to physicians’ career stages and specific age can avoid the complications that arise when a physician’s career trajectory does not correspond to his or her expectations or what is in the best interests of the medical practice plan.
